# Engineering thermoresponsive phase separated vesicles formed *via* emulsion phase transfer as a content-release platform†
†Electronic supplementary information (ESI) available. See DOI: 10.1039/c7sc04309k


**DOI:** 10.1039/c7sc04309k

**Published:** 2018-05-11

**Authors:** Kaiser Karamdad, James W. Hindley, Guido Bolognesi, Mark S. Friddin, Robert V. Law, Nicholas J. Brooks, Oscar Ces, Yuval Elani

**Affiliations:** a Department of Chemistry , Imperial College London , Exhibition Road , London , SW7 2AZ , UK . Email: o.ces@imperial.ac.uk ; Email: yuval.elani10@imperial.ac.uk; b Institute of Chemical Biology , Imperial College London , Exhibition Road , London , SW7 2AZ , UK; c Department of Chemical Engineering , Loughborough University , Loughborough , LE11 3TU , UK; d FABRICELL , Imperial College London , Exhibition Road , London , SW7 2AZ , UK

## Abstract

Giant unilamellar vesicles (GUVs) are a well-established tool for the study of membrane biophysics and are increasingly used as artificial cell models and functional units in biotechnology. This trend is driven by the development of emulsion-based generation methods such as Emulsion Phase Transfer (EPT), which facilitates the encapsulation of almost any water-soluble compounds (including biomolecules) regardless of size or charge, is compatible with droplet microfluidics, and allows GUVs with asymmetric bilayers to be assembled. However, the ability to control the composition of membranes formed *via* EPT remains an open question; this is key as composition gives rise to an array of biophysical phenomena which can be used to add functionality to membranes. Here, we evaluate the use of GUVs constructed *via* this method as a platform for phase behaviour studies and take advantage of composition-dependent features to engineer thermally-responsive GUVs. For the first time, we generate ternary GUVs (DOPC/DPPC/cholesterol) using EPT, and by compensating for the lower cholesterol incorporation efficiencies, show that these possess the full range of phase behaviour displayed by electroformed GUVs. As a demonstration of the fine control afforded by this approach, we demonstrate release of dye and peptide cargo when ternary GUVs are heated through the immiscibility transition temperature, and show that release temperature can be tuned by changing vesicle composition. We show that GUVs can be individually addressed and release triggered using a laser beam. Our findings validate EPT as a suitable method for generating phase separated vesicles and provide a valuable proof-of-concept for engineering content release functionality into individually addressable vesicles, which could have a host of applications in the development of smart synthetic biosystems.

## Abstract 2

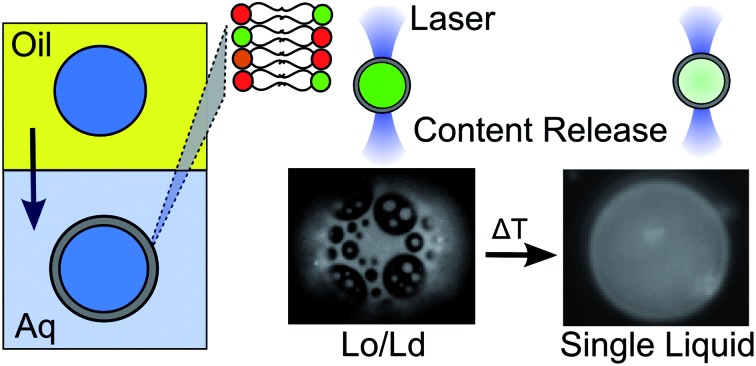
Elucidation of cholesterol insertion efficiency into phase-transfer vesicles enables the rational design of phase-separated membranes as thermally-responsive platforms for artificial cell construction.

## Introduction

Giant Unilamellar Vesicles (GUVs) are increasingly being used as functional units in biotechnology, as artificial cell chassis, as cell-mimics for the study of cellular processes, and as model membranes to study membrane biophysics.^1^–^6^,^35^ Comprised of a lipid bilayer encasing a small fluid volume, vesicles offer a biomolecular fabric for the insertion of membrane proteins while also providing a microcompartment for encapsulating material cargo. Part of their appeal is the rich phase behaviour they possess, which can be taken advantage of to add functionality, for example enabling the creation of smart and responsive systems. However, taking advantage of a membrane's inherent structural and biophysical properties is predicated on achieving accurate control of lipid composition.

A major enabler in the construction of functional GUVs has been the development of emulsion phase transfer (EPT) methods for their production,^5^,^7^–^10^ where water-in-oil droplets are used as templates around which a bilayer is assembled. This method is attractive as unlike traditional methods (such as electroformation) it allows the encapsulation of large, charged chemical species (a category which many biomolecules fall under), requires no specialised equipment, is amenable to translation into high-throughput microfluidic formats,^6^,^8^,^11^–^13^ and is uniquely capable of producing asymmetric GUVs.^14^,^15^ However, there is an open question surrounding the degree of incorporation of different amphiphiles into membranes formed by EPT, an issue which must be addressed if composition-dependent features are to be utilised, for example in the construction of vesicles with controlled surface structure.

One widely-studied membrane phenomenon is the lateral heterogeneity of membranes containing multiple lipid components. These membranes can separate into coexisting phases (or domains) that possess different degrees of molecular order and have distinct compositions.^16^ Single component membranes show a transition between a gel phase (L_β_) and fluid phase (L_α_) at a defined melting temperature (*T*_m_), a process that is associated with increased membrane permeability due to the formation of membrane defects.^17^,^18^ In two component mixtures of high- and low-melting point lipids, these phases can coexist. When cholesterol is added to form ternary mixtures, coexisting liquid disordered (L_d_) and liquid ordered (L_o_) domains can form. The L_o_ phase is enriched in cholesterol and saturated lipid (*e.g.* DPPC) and a L_d_ phase composed of mostly unsaturated lipid (*e.g.* DOPC).^19^–^21^ While both the L_o_ and L_d_ phases exhibit rapid lateral diffusion within the plane of the membrane, the lipid chains within the L_o_ phase have a significantly higher degree of order than those in the L_d_ phase. Transition from a phase separated to a mixed state can be achieved by heating above a characteristic mixing temperature (*T*_mix_) for a particular lipid composition (which can differ from the melting temperature (*T*_m_) of individual lipids).^22^

Despite being extensively studied in GUVs formed *via* electroformation, there remains a scarcity of literature on phase separation in GUVs constructed from water-in-oil emulsions. Hamada *et al.* reported the use of the EPT technique to prepare asymmetric GUVs with microdomains.^23^ However, the work merely confirmed domain formation was possible and did not probe any further. A droplet-shooting and size-filtration (DSSF) method also showed that phase separation was possible, but did not undertake a comprehensive investigation.^24^ Finally, a vesicle formation technique, ‘continuous droplet interface crossing encapsulation’ (cDICE) has recently been investigated for its potential to incorporate charged lipids and sterols.^25^ Although cDICE tolerated charged lipids well, the method was highly inefficient at incorporating cholesterol (<1% efficiency), so L_o_/L_d_ domains could not be formed.

Further investigations of the phase behaviour of GUVs formed *via* the EPT technique are needed, specifically regarding the extent to which the concentration of lipids during preparation matches that in the final GUVs. Resolving this issue is critical to assessing the suitability of this technique for unravelling fundamental principles in membrane biophysics and generating responsive micro-containers. Importantly, adding the rich membrane phase behaviour exhibited by lipid systems to the toolkit at our disposal to introduce functionality to vesicles formed *via* EPT will be vital in developing targeted, active structures. This is key given that GUVs generated *via* this method underpin many applications of GUVs as microreactors, artificial cells, responsive soft microsystems, and as tools in biotechnology.^2^,^26^–^28^,^36^

Here we characterise the cholesterol insertion efficiency of GUVs formed *via* EPT for the first time by studying the phase behaviour of ternary lipid bilayers composed of different ratios of DOPC/DPPC/cholesterol. We compare the observed phase behaviour to previous studies using electroformation to suggest a 28–50% cholesterol incorporation efficiency, but still show that the expected membrane structures can be observed using EPT GUVs by compensating for the cholesterol loss. We demonstrate that heating different ternary vesicle compositions through the *T*_mix_, either globally using a heating stage, or locally using through a laser-assisted heating setup, leads to release of encapsulated fluorophore and peptide cargo. Finally, we show that release temperature can be tuned by the user through rational design of vesicle composition. In this way we couple the encapsulation advantages associated with EPT to the phase behaviour of multi-component membranes, to engineer structurally controlled, thermoresponsive vesicles which release cargo when domains are mixed.

## Results and discussion

### The incorporation efficiency of cholesterol in EPT vesicles

**Fig. 1:**
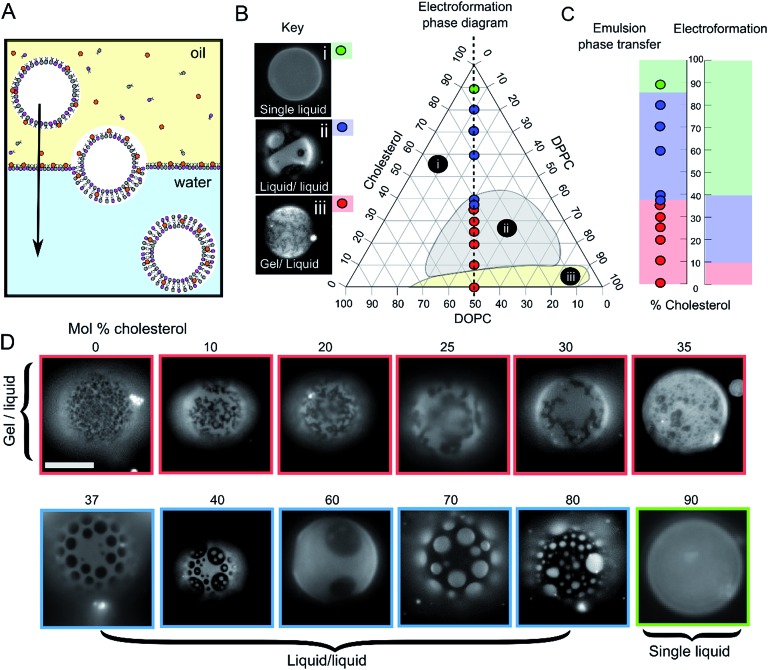
Emulsion phase transfer (EPT) can be used to produce ternary GUVs with different lateral membrane structure. (A) EPT vesicles are produced by driving lipid-monolayer stabilised water-in-oil droplets (with lipid in the oil phase) through an oil–water interface stabilised by a second lipid-monolayer. As the droplet is driven through the interface under gravity, a second monolayer is deposited, forming the final vesicle. (B) Ternary electroformation DOPC/DPPC/cholesterol phase diagram highlighting the three phase behaviours observed in GUVs: (i) a single liquid phase (green), (ii) liquid–liquid domain coexistence (blue) and (iii) gel–liquid coexistence (red). The twelve compositions studied here are overlaid on the diagram, each containing a 1 : 1 molar ratio of DOPC and DPPC, with increasing mol% cholesterol. Phase diagram is based upon the Hamada schematic.^32^ The lipid composition shown are those of the initial lipid film, and not necessarily of the GUVs themselves. (C) A comparison of ternary GUV phase behaviour between EPT (left) and electroformed vesicles (right). The observed phase behaviour is shown as a function of the mol% cholesterol in the initial lipid film. Although the same phase behaviours are observed in each case, due to reduced cholesterol incorporation, increased cholesterol is needed to reach key phase changes (∼36 *vs.* 10%, and ∼80–90 *vs.* 40% respectively). (D) Selected fluorescence microscopy images of GUVs with increasing mol% cholesterol. Each composition contains 1 mol% rhodamine-PE in order to visualise domains. In all images, the dark regions correspond to the more ordered phase (L_o_ or gel) and the light regions correspond to the L_d_ phase. Scale bar = 5 μm, experiments conducted at RT.

GUVs were formed using the centrifuge-driven EPT technique, where lipids are incorporated into the oil phase (Fig. 1A).^28^ 12 different types of GUVs were made from oils containing DOPC/DPPC (1 : 1 molar ratio) and increasing amounts of cholesterol (0–90 mol%). Incorporation of rhodamine-PE (at 1 mol% of total phospholipid concentration), which partitions into the DOPC-rich disordered phases, allowed visualization of L_d_ domains *via* fluorescence microscopy. Within these samples we observed the full range of phase behaviour observed previously in electroformed vesicles (Fig. 1B–D).

We investigated whether EPT GUVs exhibited similar phase behaviour as those prepared by electroformation, based on results obtained in previous studies.^21^ Typically, at RT electroformed vesicles with equal amounts of DOPC and DPPC phase separate into non-circular gel phase domains within a fluid continuous phase when the cholesterol mole fraction is less than 10 mol%. Between 10–45 mol% of cholesterol, L_o_/L_d_ coexistence is seen (characterised by the presence of circular fluid domains). NMR has revealed there may also be three-phase coexistence regions, although these were not observed with fluorescence microscopy.^20^ Above the 45 mol% cholesterol threshold, a single uniform liquid phase is present. This behaviour is summarised in the phase diagram in Fig. 1B and graph in Fig. 1C.

In our experiments (indicated as coloured circles and boxes Fig. 1B–D) we obtained gel/liquid phase separation in vesicles where 0–35 mol% cholesterol was present in the initial lipid-in-oil mixture. From 37–80 mol%, each vesicle composition displayed circular domains characteristic of L_o_/L_d_ phase separation. Finally, at 90 mol% cholesterol, a single liquid phase was obtained. We therefore estimate the gel/liquid to L_o_/L_d_ transition occurs between 35–37 mol% cholesterol, and the L_o_/L_d_ to single liquid transition occurring between 80–90 mol% cholesterol in the originally dissolved lipid mixture. Comparing these transitions to those obtained with electroformation gives us a cholesterol incorporation efficiency of 28% in the gel/liquid to L_o_/L_d_ transition region and 44–50% at the L_o_/L_d_ to single liquid transition region. As with electroformed GUVs, we found that increasing cholesterol concentration was associated with an increasing area fraction of dark domains in our EPT GUVs.^29^

Whilst there is clearly reduced cholesterol insertion (relative to the phospholipid insertion) in EPT vesicles compared to electroformed vesicles, the incorporation percentages reported here are significantly higher than the <1 mol% reported by Blosser *et al.* using the cDICE method.^25^ In addition, the EPT GUVs showed very low compositional dispersity (ESI Fig. SI1†), with more than 95% of vesicles generated displaying the same phase behaviour as the rest of the set (a minimum of 100 GUVs for each composition observed). This suggests that the membrane composition of EPT GUVs can be accurately controlled simply by compensating for the relative partitioning of cholesterol by adding an increased concentration to the initial lipid mixture.

To ensure that the absolute concentration of cholesterol in oil doesn't affect GUV formation, 1 : 1 : 3 DOPC : DPPC : Chol vesicles were produced from four different lipid-in-oil solutions ranging from 0.2 mg ml^–1^ to 80 mg ml^–1^ (ESI Fig. S2†). We observed L_o_/L_d_ domains in each case, despite there being a 400-fold difference in absolute cholesterol level. This demonstrates that in the concentration regime we use, absolute cholesterol concentration does not have an effect on final GUV composition. We note that below above 80 mg ml^–1^ lipid did not completely dissolve in the oil phase, and below 0.2 mg ml^–1^ vesicle yield was significantly compromised, hence these being the upper and lower bounds of the concentrations tested. The ability to generate GUVs from such low lipid concentrations bodes well for wider adoption of the phase transfer technique in downstream applications.

The cholesterol incorporation observed here is likely to be the result of cholesterol's low amphipathic character. Unlike phospholipids, which possess both a polar headgroup and lipophilic acyl chains, cholesterol is predominantly a hydrophobic molecule, which possesses only a minimal polar hydroxyl head group. This leads to an increased propensity for the cholesterol to remain dissolved in the oil phase as opposed to partitioning to the oil-water interface.

### Thermally-triggered release from phase-separated GUVs

**Fig. 2:**
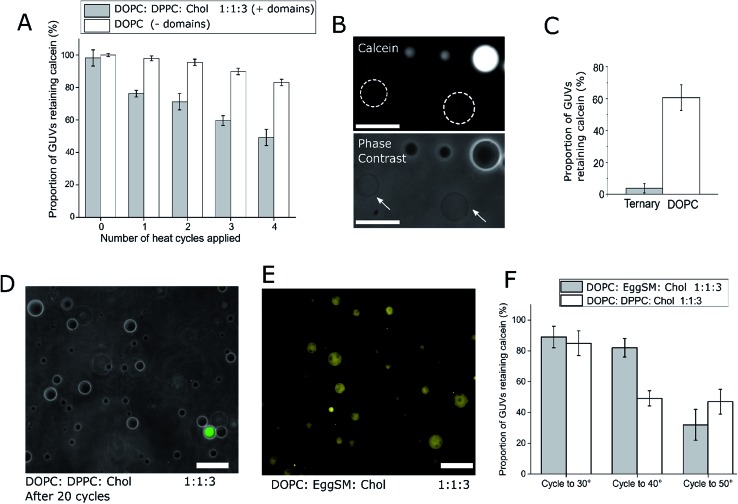
Ternary GUVs can act as a platform for user-defined, thermoresponsive content release. (A) DOPC : DPPC : Chol 1 : 1 : 3 ternary vesicles containing L_o_/L_d_ domains can release their cargo when taken through the vesicle *T*_mix_ (∼32 °C), as shown through application of multiple heat cycles and compared to domain-free DOPC GUVs. Error bars = 1 S.D., *n* = 5. (B) Fluorescence and phase contrast images highlighting calcein-free ternary GUVs after applying a heat cycle. Vesicles that leaked lost contrast during phase contrast imaging (arrows) as their internal and external content equilibrated. White dotted circles represent outlines of vesicles that have leaked scale bar = 30 μm. (C) Graph of content release after 20 thermal cycles, showing ternary GUVs underwent almost complete content release. Error bars = 1 S.D., *n* = 5. (D) Visualisation of ternary GUVs after 20 cycles with superimposed bright-field and fluorescence channels, showing the majority of vesicles released dye cargo. Scale bar = 50 μm. (E) Image of ternary DOPC : EggSM : Chol 1 : 1 : 3 vesicles successfully formed *via* phase transfer, showing the same L_o_/L_d_ domain formation. Scale bar = 50 μm. (F) Graph showing that vesicle *T*_mix_ defines the release temperature, as shown by the release of content from DOPC : DPPC : Chol (*T*_mix_ ∼ 32 °C) when cycled to 40 °C, whilst DOPC : EggSM : Chol vesicles (T_mix_ ∼ 45 °C) only release their content when cycled to 50 °C. Error bars = 1 S.D., *n* = 5.

To demonstrate the utility of EPT generated phase-separated GUVs as temperature sensitive microcontainers, we developed an assay where 1 : 1 : 3 (molar ratio) DOPC/DPPC/cholesterol GUVs were loaded with a fluorescent dye (calcein, which has an emission wavelength sufficiently spectrally separated from the rhodamine lipid to image independently). These GUVs were subjected to four temperature cycles through *T*_mix_ (>32 °C) and back down to 21 °C using a heated microscope stage and cargo release monitored (Fig. 2). This particular lipid composition was chosen as it is well studied,^21^ and has phase transition significantly above room temperature, so phase transition would only occur when actively heated.

After one cycling event, 25% of GUVs were found to have released their entire internal dye cargo. Through subsequent cycles, this proportion could be increased, with 51% of GUVs lacking calcein after four cycles (Fig. 2A). As a control, leakage through GUVs composed of DOPC (not phase separated) showed an average leakage of only 14% after 4 cycles. These statistics were obtained from 5 runs, with a minimum of 40 vesicles analyzed in each run. When vesicles leaked, they also lost contrast when imaged by phase contrast as the internal and external solutions equilibrated (Fig. 2B).

To confirm that complete calcein release can be obtained from ternary GUVs, we applied 20 heat cycles to ternary and DOPC vesicles (Fig. 2C and D). The ternary vesicle population displayed almost complete calcein release (>95% vesicles showing release), whilst ∼60% of DOPC GUVs still contained calcein, confirming the robustness of the release mechanism.

We note that in this case vesicles were incubated in 0.5 M sucrose during the thermal cycle, and subsequently transferred to 0.5 M glucose solution. For this reason, they were visible under phase contrast microscopy. Complete content release from a ternary vesicle population as shown here gives another indication to the homogeneity of the ternary vesicles formed through EPT.

Our data suggests that the bilayer leakage depends on the GUVs undergoing a phase transition, which we attribute to the biophysical phenomena of packing defects at domain interfaces.^30^ As the *T*_mix_ of this phase transition in ternary systems can be controlled through lipid composition, we also produced calcein-loaded 1 : 1 : 3 DOPC : Egg Sphingomyelin (EggSM) : Chol vesicles containing L_o_/L_d_ domains as shown in Fig. 2D. As the *T*_mix_ for this composition is *ca.* 45 °C, release should occur only when heating beyond this temperature.^21^ We therefore heat cycled both 1 : 1 : 3 DOPC : DPPC : Chol and DOPC : EggSM : Chol vesicles to 30 °C, 40 °C, and 50 °C, and monitored calcein release (Fig. 2E). As expected, the DOPC : DPPC : Chol vesicles leaked at both 40 °C and 50 °C, whereas calcein release from DOPC : EggSM : Chol vesicles only occurred after heating to 50 °C, further indicating that the *T*_mix_ of the ternary system controls content release. These results also highlight how the thermoresponsive property of ternary GUVs can be tuned by the user for use in triggered release applications.

After demonstrating the potential to tune release of a dye, we then exploited the ability of GUVs to encapsulate biomolecules. We used the model peptide biomolecule, MOCAc-RPKPVENvaWRK(Dnp)-NH_2_ (NFF-3). NFF-3 contains an N-terminal 7-methoxycoumarin fluorophore quenched *via* resonance transfer by a C-terminal 2,4-dinitrophenyl group.^37^ As the peptide contains a trypsin cleavage site between the glutamic acid and norvaline residues, proteolysis of NFF-3 results in liberation of fluorescence, which can be monitored spectroscopically (ESI Fig. S3A†). We used the fluorogenic nature of NFF-3 to design a peptide release assay, where the leakage of peptide can be monitored through adding trypsin to the external solution (ESI Fig. S3B†). One heat cycle was applied to peptide-loaded 1 : 1 : 3 DOPC : DPPC : Chol and DOPC vesicles. Trypsin was then added to external solution, and the change in fluorescence quantified against untreated vesicle controls. As shown in ESI Fig. S2C,† appreciable peptide release from ternary vesicles was observed (22%, S.D. = 7%, *n* = 9), whilst negligible release occurred from DOPC vesicles (2.5%, S.D. = 4%, *n* = 4).

One important property of the release system that is still to be characterised is the size limit of the transient pores generated in ternary vesicles. Although packing defects in dimyristoylphosphatidylcholine vesicles with an effective molecular weight cut-off of 900 Da have been recorded previously,^30^ here we show release of peptide molecules with a weight of 1674 Da. This release may be due to partial unfolding of the linear polypeptide chain of NFF-3 during cargo release (resulting in smaller effective molecular weight), or alternatively, indicate that the packing defects generated in ternary systems possess larger sizes compared to gel–fluid transitions observed in single lipid systems.

Using heat to trigger leakage in L_o_/L_d_ ternary vesicles represents a proof-of-concept that could be applied to a host of applications involving the controlled release of material; however, one drawback to the methods employed above is that the response of bulk GUV populations was monitored, as opposed to individual GUVs.

### Phase changes and cargo release in individual GUVs through laser-induced local heating

**Fig. 3:**
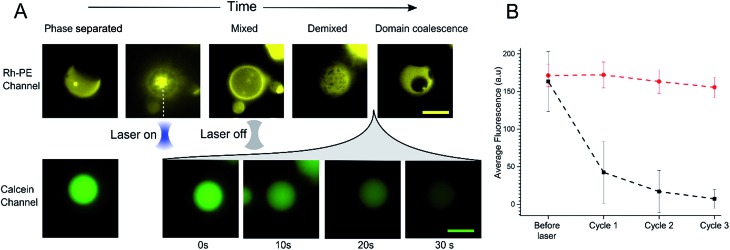
(A) Image sequence showing the heating process for a DOPC : DPPC : Chol 1 : 1 : 3 phase separated GUV using an optical trap. Once the laser was turned on the sample was locally heated and the ordered and disordered liquid domains mix into a uniform state (above the *T*_m_ ∼ 32 °C). After this point the laser was switched off to allow the GUV to cool and the domains to demix and eventually coalesce. Imaging the same vesicles in the calcein channel revealed leakage once the GUV was heated above *T*_m_ and allow to cool (∼30 seconds). Scale bars = 10 μm. (B) Graph showing average mean fluorescence of phase separated GUVs (black squares) or single phase DOPC GUVs (red circles) (diameter 7–12 μm) after successive cycling events. Error bars = 1 S.D., *n* = 10.

In order to further investigate the phenomenon of content release, we induced phase changes in selected vesicles by employing a tightly focused laser beam from an optical tweezers set-up to locally heat individual GUVs. This was achieved by using a single-beam optical trap, which locally heats each targeted vesicle. We applied a 1.1 W (at trap) infrared laser to individual selected phase separated GUV. This leads to an increase in temperature which was sufficient to raise the GUV above the *T*_mix_ (estimated temperature increase due to laser ∼21 °C)^38^ as shown in Fig. 3A. Upon applying the laser, the domains mixed to form a uniformly fluorescent liquid phase within 5 seconds. Once in the mixed phase, the trap was switched off to allow the vesicle to cool, leading to immediate demixing and the reappearance of coexisting liquid phases.

While monitoring the rapid transition between coexisting and uniform phases, fluorescent images were taken in the calcein channel to monitor the fluorescence of the GUV internal content. Between being in a mixed state and demixing into separate coexisting liquid phases release of calcein was observed (Fig. 3A). This release process was monitored over a period of 30 seconds; during this time, L_o_ domains were coalescing to form larger domains. Multiple cycles of laser-induced localised heating were then carried out on individual GUVs with or without domains (Fig. 3B). After a single cycle, the internal fluorescence of phase separated GUVs decreased by ∼66% (*n* = 10), whilst the fluorescence of single-phase GUVs showed no change. Two further heating cycles were then performed, and after the third cycle, the phase separated GUVs showed almost complete loss of fluorescence, whilst minimal change was observed inside single phase vesicles.

As with bulk GUV populations, we hypothesise that leakage is due to packing defects along the increased domain line interface during the phase transition. When GUVs were fully phase separated into two distinct L_o_ and L_d_ phases (half-moon topology) the domain line interface is relatively low when to compared with the demixing domain ‘ripening’ stage. During this stage, where many nano/microdomains are growing through collision and coalescence, the line interface between domains at its highest. Packing defects between domain boundaries are thus most prominent here which leads to the formation of transient gaps between the domains through which material can leak out. This phenomenon has been reported previously in gel/liquid systems.^18^,^31^,^39^,^40^

## Conclusions

We have shown that GUVs made using EPT exhibit the full range of phase behaviour observed in electroformed GUVs and can be used as thermosensitive microcompartments that can be individually targeted and triggered to release their cargo. We detail the phenomenon of membrane leakage when coexisting fluid membranes are taken through the *T*_mix_ and back. Previous work in this area has focused on single component systems that transition between gel and fluid phases. To our knowledge, this is the first work to confirm that any emulsion-based method can replicate the full range of phase behaviour previously displayed in ternary vesicles,^24^,^25^,^32^ and the first time that a laser has been used to induce phase transitions and content release in phase separated GUVs. Vesicles that display lateral phase separation offer advantages over single component systems for cargo release applications in that the transition temperature (and thus release temperature) can be finely controlled by tuning the lipid composition, as shown through the replacement of the saturated DPPC lipid with EggSM (Fig. 2). We demonstrated the versatility of our release platform through the encapsulation and triggered the release of both the fluorescent dye calcein as well as the fluorogenic peptide NFF-3.

A second key advantage of using ternary systems lies in the high level of structural control. Membrane organisation could serve as a foundation for controlled patterning of membrane-bound molecules in sensing or communication applications; by employing membrane phase separation, the transition temperature becomes a switch not just for content release, but also the controlled mixing of membrane-bound species anchored to different domains. These switchable systems could also be exploited in responsive cell-mimics where they can behave as thermal transducers to initiate down-stream chemical events within an artificial cell.

## Materials and methods

### GUV generation *via* emulsion phase transfer

DOPC, DPPC, rhodamine-PE were purchased from Avanti Polar Lipids. All other reagents were purchased from Sigma Aldrich. All experiments were performed in DI water and at 21 °C unless otherwise stated. A protocol for the EPT method was developed based upon previous methods reported in the literature.^7^,^28^,^33^ DOPC/DPPC/cholesterol lipids films containing 1 phospholipid mol% rhodamine-PE were made by first dissolving lipids in chloroform at appropriate ratios, followed by removal of chloroform under a stream of nitrogen and placing under vacuum for a minimum of 60 minutes. The lipid film was dissolved in mineral oil (2 mg ml^–1^) by sonicating for 30 minutes at 40 °C. An emulsion of droplets in oil was made by vortexing 20 μL of a 0.5 M sucrose aqueous solution in 200 μL of lipid-in-oil for 20 seconds. In calcein loaded-GUV experiments aqueous solution also contained 1 mM calcein. This solution was then layered above 250 μL of 0.5 M glucose in an Eppendorf to form a water/oil column, with an emulsion present in the oil phase. Droplets were driven through the interface to form GUVs by centrifugation at 9000*g* for 30 minutes, resulting in a pellet at the bottom of the tube. The supernatant was extracted and the pellet resuspended in 0.5 M glucose by pipetting up and down five times.

### Optical and fluorescence microscopy of GUVs

Vesicles were visualised with a Nikon Eclipse TE2000-E inverted microscope, and recorded on an Andor Zyla, sCMOS based camera. During fluorescence imaging samples were illuminated with a mercury arc lamp, the TRITC and FITC filter were used to visualise fluorescent rhodamine-PE (200 ms exposure) and encapsulated calcein dye (100 ms exposure) respectively. Fluorescence exposure was kept at a minimum to minimise photo-oxidation of unsaturated DOPC lipid.

### Thermally responsive dye release

Thermally responsive dye release assays were conducted on GUVs formed from 1 : 1 : 3 DOPC : DPPC : cholesterol or DOPC : EggSM : cholesterol lipid film. 20 μL of GUV solution was placed between two cover slips, which was in turn placed on a water bath controlled heating stage located on the microscope. Vesicles were cycled back and forth through the L_o_/L_d_ phase transition temperature and calcein release observed.

When vesicles were taken through 20 heat cycles to achieve full content release, this was done in bulk, by placing an Eppendorf containing vesicles on a heating rack placed on a hot plate set at 40 °C. The sample was left for 5 minutes, then removed and allowed to cool back to RT for 5 further minutes. This was repeated 20 times. In these experiments, vesicles contained 0.5 M sucrose both internally and externally, so they would not sediment. They were then transferred to the microscope for viewing, where they were diluted 1 : 9 in 0.5 M glucose in order for them to settle on the glass slide.

In the laser-induced heating experiments, 20 μL of GUV solution was placed on a Nikon TE2000-U inverted epi-fluorescence microscope combined with a custom optical trapping system. The latter has been described and characterised in previous publications^34^ and comprises a single Gaussian beam from an Ytterbium fibre laser source (20 W at 1070 nm) which is directed to the microscope objective (60× 1.4 NA) through a custom optical train. Individual vesicles were heated past the phase transition by applying a focused laser to the centre of the vesicle for 5 seconds.

### Triggered release of peptide biomolecules

MOCAc-RPKPVENvaWRK(Dnp)-NH_2_ (NFF-3) peptide was encapsulated (15 μM) within 1 : 1 : 3 DOPC : DPPC : Chol GUVs produced as detailed above. An additional purification step was added when preparing these vesicles; after resuspension of the vesicle pellet in 0.5 M glucose, the suspension was centrifuged at 6000*g* for 10 minutes, before resuspension in 0.5 M sucrose to avoid gradual sedimentation of the vesicles during spectroscopic measurements. Vesicles were exposed to a single heat cycle consisting of heating at 40 °C for 15 minutes before leaving the vesicles at room temperature for 30 minutes. TPCK-treated bovine trypsin (T1426 SIGMA, Sigma-Aldrich) was then added to external solution (0.1 μM, prepared in 40 mM HEPES, 0.5 M sucrose pH 7.4) and the fluorescence emission of proteolysed NFF-3 recorded (*λ*_ex/em_ = 320/405 nm) using a Horiba Jobin-Yvon Fluoromax 4 Spectrofluorometer (Horiba, Japan). Fluorescence emission was compared against the emission of freshly prepared vesicle samples with added trypsin (used as a control to obtain background levels of fluorescence of free peptide in solution). All vesicles samples were then lysed by adding Triton X-100 detergent to a final concentration of 0.03 v/v%, mixing and leaving for 1 hour. Then, fluorescence emission was then recorded again to obtain values corresponding to ‘100% leakage’. In order to calculate the percentage of peptide leakage, the raw data was processed as follows:
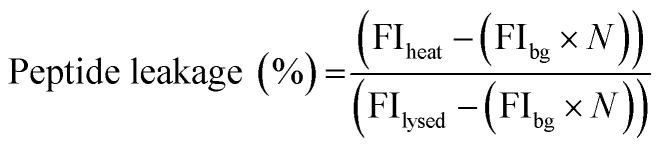
where FI_heat_, FI_bg_ and FI_lysed_ are the fluorescence intensities of the peptide sample after heating, the background fluorescence intensity derived from the control sample and the fluorescence intensity of the heat cycled sample after lysis with Triton X-100. *N* is the normalisation factor:
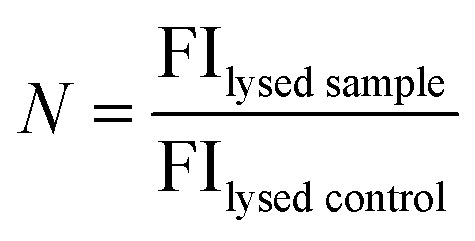
where FI_lysed sample_ and FI_lysed control_ are the fluorescence intensity of the sample and the control after lysis with Triton X-100. *N* takes into account small differences in peptide concentration in the cuvettes between the control and sample. *N* can be determined in this manner due to the linear relationship between fluorescence intensity and peptide concentration in the concentration range used in these experiments (ESI Fig. S4†).

## Conflicts of interest

There are no conflicts to declare.

## Supplementary Material

Supplementary information
