# Supplementary material for: Engineering thermoresponsive phase separated vesicles formed via emulsion phase transfer as a content-release platform
Source: Chem Sci. 2018 May 11;9(21):4851–8. doi: 10.1039/c7sc04309k (PMC5982195; doi:10.1039/c7sc04309k)
Supplement: Supplementary file 1 [file SC-009-C7SC04309K-s001.pdf]

Electronic Supplementary Material (ESI) for Chemical Science.
This journal is © The Royal Society of Chemistry 2018

Supplementary Information

Engineering thermoresponsive phase separated vesicles formed via emulsion phase transfer as a content-release platform

Kaiser Karamdad[ab†], James W. Hindley[ab†], Guido Bolognesi[c], Mark S. Friddin[a], Robert V. Law [ab], Nicholas J. Brooks[ab] ,Oscar Ces[ab]* and Yuval Elani[ab]*

Supplementary Figures

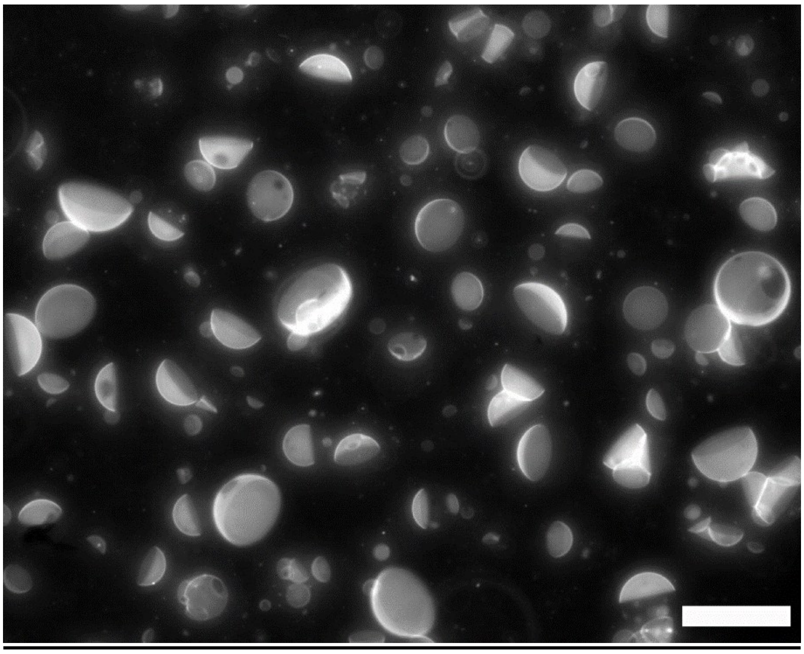

Figure S1. Representative fluorescence image of GUV population formed via emulsion phase transfer showing uniform domain morphology. Composition = DOPC: DPPC: Chol, 1:1:3. Scale bar = 20 µm.

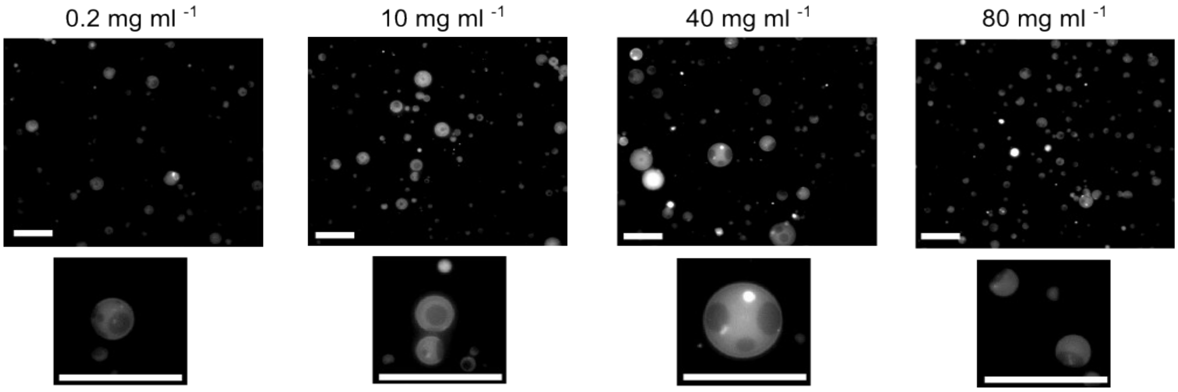

Figure S2. Effect of changing absolute concentration of lipid-in-oil on domains in GUVs formed via emulsion phase transfer. Fluorescence microscopy images of DOPC:DPPC:Chol 1:1:3 vesicles at different total lipid concentrations are shown. L_o/L_d domains were seen at all concentrations. Images below are zoomed in areas of the images above. Scale bar for all images = 50 µm.

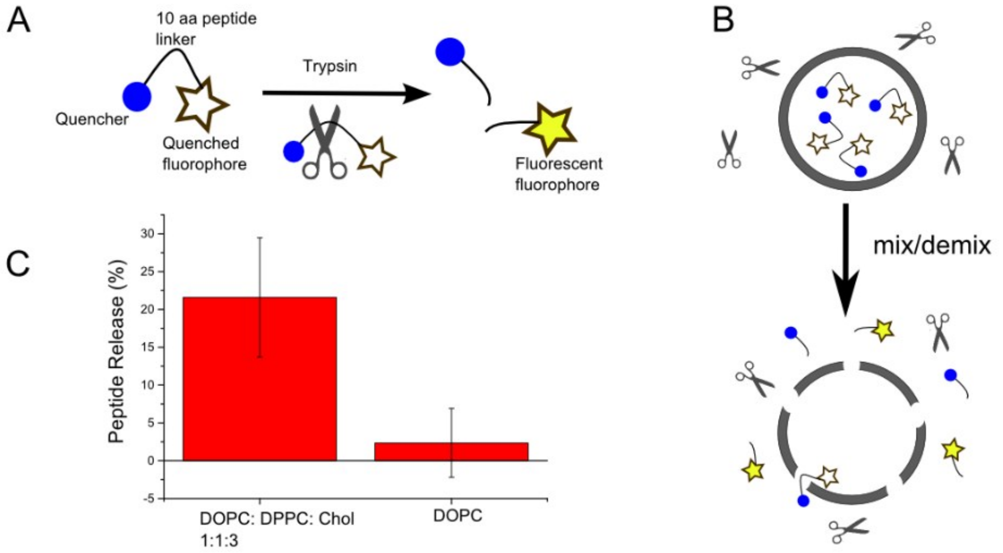

Figure S3. Peptide molecules can be released from ternary GUVs. (A) NFF-3 is a fluorogenic peptide that can be cleaved by the protease trypsin. An N-terminal fluorophore is quenched via FRET by a C-terminal dinitrophenol group, and upon proteolysis a fluorescent signal is generated. **(B)** The fluorogenic properties of NFF-3 enables the design of an enzymatic leakage assay, where peptide-loaded GUVs are taken through a heating cycle. If peptide can escape during the mix/demix cycle, trypsin present in external solution can proteolyse the peptide, generating signal that can be monitored. **(C)** Peptide release can be triggered from ternary vesicles by applying a heating cycle. Minimal release is observed in DOPC vesicles lacking such domain structure. Errors bars represent 1 s.d. n=9 for ternary vesicles, n=4 for DOPC vesicles.

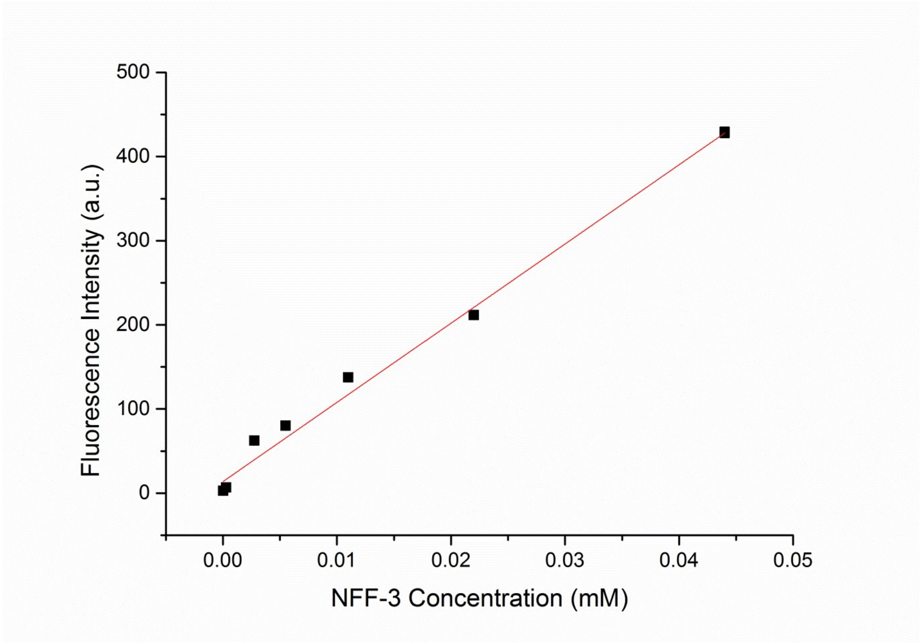

Figure S4. NFF-3 fluorescence Calibration Curve. Within the concentration range used in the peptide release experiments above (0.015 mM) , NFF-3 fluorescence varies linearly with concentration. Error bars represent 1 s.d., n=3, r^2= 0.99667.
